# Predator–prey interactions in a ladybeetle–aphid system depend on spatial scale

**DOI:** 10.1002/ece3.4117

**Published:** 2018-06-11

**Authors:** Wei‐Ting Lin, Steven C. Pennings

**Affiliations:** ^1^ Department of Biology and Biochemistry University of Houston Houston Texas USA

**Keywords:** *Aphids*, *Coccinellids*, *Cycloneda sanguinea*, *Iva frutescens*, metacommunity, scale‐dependent, spatial scale, species interaction, *Uroleucon ambrosiae*

## Abstract

The outcome of species interactions may manifest differently at different spatial scales; therefore, our interpretation of observed interactions will depend on the scale at which observations are made. For example, in ladybeetle–aphid systems, the results from small‐scale cage experiments usually cannot be extrapolated to landscape‐scale field observations. To understand how ladybeetle–aphid interactions change across spatial scales, we evaluated predator–prey interactions in an experimental system. The experimental habitat consisted of 81 potted plants and was manipulated to facilitate analysis across four spatial scales. We also simulated a spatially explicit metacommunity model parallel to the experiment. In the experiment, we found that the negative effect of ladybeetles on aphids decreased with increasing spatial scales. This pattern can be explained by ladybeetles strongly suppressing aphids at small scales, but not colonizing distant patches fast enough to suppress aphids at larger scales. In the experiment, the positive effects of aphids on ladybeetles were strongest at three‐plant scale. In a model scenario where predators did not have demographic dynamics, we found, consistent with the experiment, that both the effects of ladybeetles on aphids and the effects of aphids on ladybeetles decreased with increasing spatial scales. These patterns suggest that dispersal was the primary cause of ladybeetle population dynamics in our experiment: aphids increased ladybeetle numbers at smaller scales because ladybeetles stayed in a patch longer and performed area‐restricted searches after encountering aphids; these behaviors did not affect ladybeetle numbers at larger spatial scales. The parallel experimental and model results illustrate how predator–prey interactions can change across spatial scales, suggesting that our interpretation of observed predator–prey dynamics would differ if observations were made at different scales. This study demonstrates how studying ecological interactions at a range of scales can help link the results of small‐scale ecological experiments to landscape‐scale ecological problems.

## 1 INTRODUCTION

Patterns of population dynamics are usually spatial scale dependent: Study outcomes might depend on the scale at which observations and measurements are made (Levin, 1992; Wiens, 1989). Although species interactions usually occur at relatively small spatial scales, the consequences of species interactions may be more manifest at larger spatial scales due to the dispersal of organisms (e.g., Bach, 1980; Seitz, Lipcius, & Hines, 2017). The issue of scale dependency is most pressing for experimental studies. Although experiments provide powerful insight into species interactions, they are usually conducted at a single, arbitrary spatial, and temporal scale (Horne & Schneider, 1995; Wheatley & Johnson, 2009) and fail to adequately incorporate patterns of species dispersal. It is desirable to scale up local observations to address larger scale ecological problems, such as how environmental changes may affect landscape‐scale ecological patterns (Chave, 2013; Chesson, 2012). However, the effectiveness of the “scaling up,” or extrapolation endeavor can easily be compromised by the scale dependency of population dynamics (Hunsicker et al., 2011; Miller, Turner, Smithwick, Dent, & Stanley, 2004).

In food web modules that involve predator–prey interactions, the scaling up process is further complicated by the distinct life‐history traits of the interacting species. Predator and prey species usually differ in body size and dispersal ability and thus experience the environment at different spatial resolutions (Roland & Taylor, 1997). Moreover, because many life‐history traits scale with body size (Lawton, 1990; Lindstedt & Calder, 1981), predator and prey species will have different colonization and extinction dynamics in local patches. Therefore, local food–web interactions do not simply dictate population dynamics at larger scales. Theoretical analyses of metacommunities have shown that dispersal of organisms among patches of suitable habitat can promote the persistence of predator–prey modules that, if trapped in a single patch, would crash (Briggs & Hoopes, 2004; Taylor, 1990). Experimental and observational studies have also shown that the spatial scale of the experimental system and habitat grain can be critical for the coexistence of competitors, and the persistence and structure of predator–prey systems (Holyoak & Lawler, 1996; Huffaker, 1958; Janssen, van Gool, Lingeman, Jacas, & van de Klashorst, 1997). For example, populations of Atlantic cod only correlated with their prey (capelins) at scales larger than 15 km, when capelins were aggregated at a thermal refuge (Rose & Leggett, 1990). Using spatial cross‐correlation methods, Fauchald, Erikstad, and Skarsfjord (2000) found that seabirds search for fish patches at two characteristic scales (~50 km and ~3 km). In a crab‐clam system, Seitz and Lipcius (2001) found that the top‐down effect of crabs on clams was important at small scales (~5 km long river) but not at larger scales (~50 km). These and other studies have shown that analyses conducted at different spatial scales can lead to different conclusions, emphasizing the importance of explicitly including scale in studies of species interactions.

Here we consider the issue of spatial scale using an aphid‐ladybeetle system. Aphids and their coccinellid predators are a model system for studying biological control and predator–prey interaction dynamics (Dixon, 2000). Spatial refuges are often important in aphid population dynamics (Gonzáles, Gianoli, & Niemeyer, 2001; Hacker & Bertness, 1995; Hopkins & Dixon, 1997), suggesting that important aspects of ladybeetle–aphid interactions manifest at larger spatial scales that include both patches with predators and refuge patches that lack predators. Aphid populations often possess strong spatial turnover (e.g., Weisser & Härri, 2005), also indicating that the colonization–extinction processes emphasized in metapopulation models may be important for their population dynamics. Despite the strong impact of individual ladybeetles on individual aphid colonies (Minoretti & Weisser, 2000), the effectiveness of ladybeetles in suppressing aphid populations at large spatial scales is variable (Kindlmann, Yasuda, Sato, Kajita, & Dixon, 2015). These different lines of evidence suggest that some ladybeetle–aphid systems might persist at large spatial scales as metacommunities consisting of patches with and without predators, and that results from individual patches cannot be scaled up to the landscape without understanding the metacommunity dynamics.

At the landscape scale, the negative impacts of ladybeetles on aphids are related to the ability of ladybeetles to find and aggregate at dense patches of aphids (Costamagna & Landis, 2011; Kareiva, 1987). Ladybeetles can find appropriate habitat patches from a distance using visual cues; however, the ability of ladybeetles to visually detect the presence of aphids is limited to a very small spatial scale (<7 mm) (Nakamuta, 1984). When ladybeetles encounter aphids, they tend to stay longer at a patch (Ives, Kareiva, & Perry, 1993) or perform area‐restricted searches (Kareiva & Odell, 1987). These behaviors promote the aggregation of ladybeetles at patches with more aphids and create a positive relationship between aphid densities and the change in ladybeetle densities. However, this aggregation is not always found in the field, as a number of studies have found that the spatial distribution of ladybeetles at large scales does not track the distribution of aphids very well (Hacker & Bertness, 1995; Krivan, 2008; Kummel, Brown, & Bruder, 2013). Therefore, in ladybeetle–aphid systems, it seems that both the effect of the predator on the prey and the effect of the prey on the predator potentially vary with spatial scale. Understanding the scale dependency of ladybeetle–aphid interactions would help us better interpret observations of this and similar predator–prey systems, and better scale up small‐scale experimental results to larger scale patterns.

To understand the metacommunity dynamics of ladybeetles and aphids in their natural habitat, we studied time series data of their populations at two spatial scales. We calculated their interaction strength at the scale of each patch, and we calculated the patch occupancy and colonization rates at the metacommunity scale. To further study how the interaction between ladybeetles and aphids changes with spatial scale, we evaluated the effects of ladybeetles on aphids and the effects of aphids on ladybeetles in an experimental system where the habitat (host plants of the specialist aphids) was manipulated to facilitate analysis across multiple spatial scales. To better understand the mechanisms driving the observed species abundance patterns, and to explore the generality of the results, we also built a spatially explicit metacommunity model parallel to the experimental system. We hypothesized that (*H1*) ladybeetles have stronger negative effects on aphids at smaller scales, and weaker effects at larger scales. We reasoned that although ladybeetle predators would suppress local aphid populations by feeding and induction of aphid dispersal, ladybeetles might not control aphid populations as effectively at larger scales because aphids can multiply on predator‐free patches. We further hypothesized that (*H2*) aphids have stronger apparent positive effects on ladybeetles at smaller scales, due to the adaptive movement of ladybeetles. We reasoned that because both longer patch layover time and area‐restricted searching by ladybeetles are triggered by physical encounters with aphids, ladybeetle population dynamics should respond to aphid population sizes at smaller scales. In our experiment, we monitored short‐term population dynamics at a fine (daily) temporal grain and focused on within‐season dynamics; therefore, we assumed that ladybeetle growth and death had little effect on their population dynamics. We tested these two hypotheses with the field experiment and the metacommunity model. For the model, we also simulated an additional model scenario that included predator demographic processes to explore how this would change model outcomes.

## 2 MATERIALS AND METHODS

### 2.1 The plant–aphid–ladybeetle system

**Figure 1:**
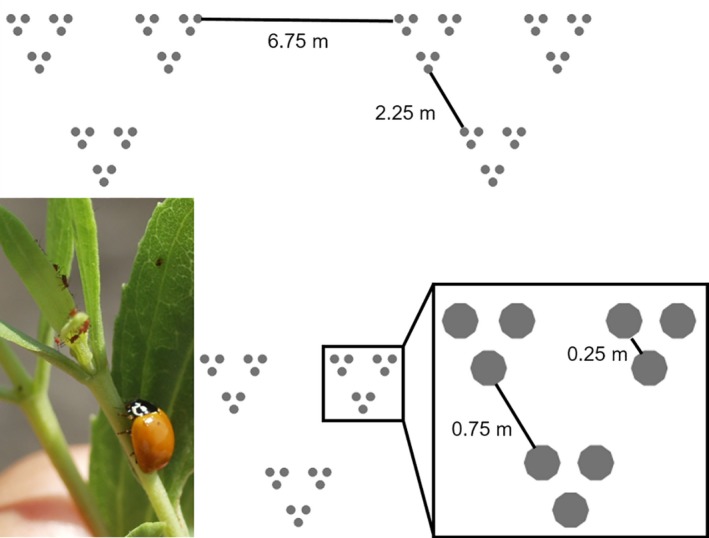
Field experiment. The field experiment contained 81 potted *Iva* plants (gray circles), set up in a hierarchical spatial array. The inset panel shows a nine‐plant set. The distance between pots is marked in the figure. The diameter of a pot is about 0.25 m. The picture shows the study species: the host plant, marsh elder (*Iva frutescens*), aphids (*Uroleucon ambrosiae*), and a spotless ladybeetle (*Cycloneda sanguinea*)

Field work was conducted in the Sapelo Island National Estuarine Research Reserve on Sapelo Island, Georgia, USA (31°24ʹ18ʺ N, 81°16ʹ32ʺ W). Marsh elder (*Iva frutescens,* hereafter *Iva*) is a shrub found at the terrestrial border of the high marsh. Insect food web modules on *Iva* have been the focus of a number of previous studies of predator–prey interactions (Hacker & Bertness, 1995; Ho & Pennings, 2008; Marczak et al., 2011). We studied a simple system consisting of the most common herbivore on *Iva*, the aphid *Uroleucon ambrosiae* (hereafter *Uroleucon*) (Hacker & Bertness, 1995), and its major predator at our study site, the spotless ladybeetle *Cycloneda sanguinea* (hereafter *Cycloneda*, Figure 1). Other predators of *Uroleucon* at this site include other ladybeetles such as *Coccinella septempunctata*,* Harmonia axyridis,* and *Hippodamia convergens*, and an omnivorous crab *Armases cinereum* (Ho & Pennings, 2008; Marczak et al., 2011; Pennings et al., 2009).

### 2.2 Field observations

To document natural interactions between ladybeetles and aphids in the field, we visually sampled the insect communities on 38 patches of *Iva* every 3 days for a total of 59 days (19–20 samples from each patch) in the summer of 2014. Each patch consisted of one or more adjacent *Iva* plants with contiguous canopies; patches were separated from one another and from other *Iva* plants by at least 0.5 m. Adult and larval ladybeetles of *Cycloneda sanguinea*,* Coccinella septempunctata* and *Naemia* sp. were lumped as “ladybeetles” in the analysis. We calculated the effect of ladybeetles on aphids and the effect of aphids on ladybeetles at the scale of each patch (see below, Section 2.5).

To study the metacommunity dynamics, we then calculated the average colonization rates over all the intervals. Colonization events of ladybeetles (or aphids) could be detected when ladybeetles (or aphids) were absent in a patch on one date and present on the subsequent date. For each 3‐day interval, the colonization rate was calculated as the number of colonization events divided by the number of empty patches at the beginning of that interval.

### 2.3 Field experiment

The field experiment was designed to assess the importance of spatial scale on the ladybeetle–aphid interaction. *Iva* plants were propagated from cuttings collected in September 2013 in pots in a greenhouse adjacent to the marsh. In May 2014, we placed 81 potted plants (88.9 ± 1.03 (SE) cm tall, with 141 ± 3.9 (SE) leaves) in a fractal‐designed spatial array (Figure 1). These potted plants were smaller than most plants studied in the field, but were comparable in size to ones used in previous mesocosm experiments studying *Iva* arthropod food webs (Ho & Pennings, 2008; Marczak et al., 2011). The plants were set up in a grassy field (31°24ʹ18ʺ N, 81°16ʹ32ʺ W), about 500 m away from the marsh so that omnivorous marsh crabs (*Armases cinereum*) would have little access to the plants (none were observed at the experimental site during the study). Six additional pots of smaller *Iva* plants were put in the center of the array to increase the overall habitat attractiveness, but these plants were not sampled. This array allowed analysis at five spatial scales, consisting of 1, 3, 9, 27, and 81 plants. Although habitat fragmentation, between‐patch distance and system area all may affect the dispersal behavior and distribution of ladybeetles (Grez, Zaviezo, & Rios, 2005; Grez, Zaviezo, Tischendorf, & Fahrig, 2004; Holt, 1985; Zaviezo, Grez, Estades, & Perez, 2006), we did not seek to investigate their isolated effects. Instead, the fractal design of our experiment allowed a systematic increase in system area, average between‐patch distance, and total patch size with increasing spatial scale and has been regarded as one neutral way to simulate complex habitats (Halley et al., 2004).

When the experiment started (early‐May 2014), the plants had already been naturally colonized by aphids. Because the greenhouse was adjacent to the marsh, we assumed aphid densities were natural, and we did not manipulate the aphid populations further. We stocked the experiment with field‐captured *Cycloneda* ladybeetles and their first‐generation laboratory descendants. We used the field occupancy rate from a pilot survey (0.106 ladybeetles/plant) to stock ladybeetles in the field experiment, with the caveat that the potted plants were smaller than those sampled in the field. One day before we started recording data, we placed one ladybeetle on each of nine haphazardly chosen plants of the array. Every day during the experiment, two additional ladybeetles were put onto the plants in the center of the array to partially compensate for emigration, which we assumed was higher than natural because the experimental plants were isolated from natural, more extensive stands of *Iva* plants. We recorded the number of aphids and ladybeetles on each plant daily. If the total population of ladybeetles dropped to zero, or to one for two consecutive days, we re‐stocked the ladybeetles and started a new experimental round. Between 17 May and 26 June 2014, we conducted four rounds of the experiment, for a total of 34 study days. The full data of *Cycloneda* ladybeetle and aphid population time series can be found in Appendix S1 in Supporting Information.

Because the experiment was not enclosed in a cage, both the target species and other arthropods could disperse in and out of the system. In practice, ladybeetles tended to disperse out of the system, creating an overall decline in ladybeetle population density through each round of the experiment. We statistically corrected for this trend by simulating null models with the same temporal trend. We also recorded the number of immigrant predators (spiders and ladybeetle other than *Cycloneda*) each day when we collected data.

### 2.4 Mathematical model

We used a stochastic metacommunity model to simulate the ladybeetle–aphid system in a habitat of 81 patches arranged in a triangular array similar to the experimental system. Herbivore (aphid) and predator (ladybeetle) populations (in terms of biomass) on patch *i* at time *t* were described as *H*_*i,t*_, *P*_*i,t*_, respectively. Herbivore biomass was described by logistic growth, predation, and density‐independent mortality. We assumed a type II functional response as found in other ladybeetles and aphid species (Kummel et al., 2013; Pervez & Omkar, 2005).

(1) $$\left\{\begin{array}{c} H_{i,t+1}=H_{i,t}+g_{H}H_{i,t}\frac{k_{H}-H_{i,t}}{k_{H}}\frac{e_{P}H_{i,t}P_{i,t}}{H_{i,t}+H_{0}}-m_{H,i,t}H_{i,t}\\ P_{i,t+1}=P_{i,t}+a_{P}\frac{e_{P}H_{i,t}P_{i,t}}{H_{i,t}+H_{0}}-m_{P,i,t}P_{i,t} \end{array}\right.$$

Here, *g*_*H*_ is the rescaled intrinsic growth rate, and *k*_H_ is the carrying capacity of the herbivore, *e*_*P*_ is the predation rate, *H*_0_ is the half saturation density and *a*_P_ is the assimilation rate, *m*_*P,i,t*_ and *m*_*H,i,t*_ are the density‐independent per capita mortality rates of the predator and herbivore, respectively. The local population dynamics were described in terms of ordinary difference equations and then translated into probabilities of stochastic events as described below.

We simulated the model with two scenarios for predators. In scenario (*i*), the predator did not have demographic dynamics (Equation (1), but with *P*_*i, t*+1_ = *P*_*i,t*_). This scenario focused on short‐term dynamics of the ladybeetle–aphid system and is better aligned with our field experiment, where we did not observe a second generation of ladybeetle adults (Appendix S3). In scenario (ii), the predator had demographic processes (Equation (1)). This scenario represents more general cases of predator–prey interactions.

Emigration rates of predator and herbivore were both affected by local herbivore density, but in opposite ways. For aphids, we assumed the emigration rate increased if local aphid population density exceeded a threshold. Crowding is known as a major cue for aphids to produce alate (winged) offspring (Dixon, 1985; Hille Ris Lambers, 1966) and for alates to disperse (Walters & Dixon, 1982). For ladybeetles, we assumed the emigration rate increased when local aphid population dropped below a threshold. Ladybeetles prolong patch retention and perform area‐restricted searches after consumption of or encounter with aphids (Kareiva & Odell, 1987). We assumed that emigration was independent of patch location, although spatial configuration was found to affect ladybeetle dispersal in other studies (Grez & Prado, 2000). We applied a logistic smoothing function to make the dispersal more realistic.

We assumed that emigrating herbivores and predators settled on patches indiscriminately. Immigration of ladybeetles was found to be independent of aphid densities (Cardinale, Weis, Forbes, Tilmon, & Ives, 2006), and a model with unconditional immigration of the ladybeetle produced population distribution patterns that fit best with field data (Krivan, 2008). The probability that each emigrant from patch *i* would settle at patch *j* depended on the distance between patches *i*,* j* and was modeled as an incidence function (Hanski & Woiwod, 1993). By implementing informed emigration and random immigration, species in our model performed informed, but not optimal habitat selection. In theory, less‐than‐optimal dispersal can decrease the effectiveness of predators in suppressing prey at the landscape scale (Holt, 1985); while informed dispersal would result in higher aggregation of predators (Rubin, Ellner, Kessler, & Morrell, 2015).

We assumed that the birth, death, and dispersal of the two species were Poisson's stochastic events, with the probabilities of each event described by the ordinary difference equations (Equation (1) and equations in Appendix S2). We simulated the model with Gillespie's algorithm (Gillespie, 1976), for 100 units of time (days) and we recorded the populations once per day over the last 10 days (at *t* = 91, 92…100). The simulation was run 300 times with random initial values for herbivore and predator densities. In this way, we minimized the effects of initial population densities and generated a population time series dataset similar in structure to the experimental data (see Appendix S2 for details of the model).

### 2.5 Data analysis

The same statistical method was used to analyze the data from the field experiment and the model simulation. Both experiment and model produced population time series data for predators and herbivores at each plant (patch). Time series data at larger spatial scales were generated by summing up the data for multiple patches. We calculated the per capita effect of one species on the other for each time series, and used this per capita effect as the indicator of species interaction strength. The effect of ladybeetles on aphids, denoted *R*_TD_ (“top‐down”), was calculated as the coefficient (R) of linear regression between the decrease in the aphid population (the log ratio: log(*H*_*t*_ + 1) − log(*H*_*t*+1_ + 1)) and the mean ladybeetle population (*P*_*t*_ **+ *P*_*t*+1)_/2. The effect of aphids on ladybeetles, denoted *R*_BU_, (“bottom‐up”), was calculated as the coefficient (R) of the regression between the increase in the ladybeetle population (the log ratio: log(*P*_*t+1*_ + 1) *−* log(*P*_*t*_ **+ 1)) and the mean aphid population (*H*_*t*_ **+ *H*_*t*+1)_/2. Data points where both species were absent (*P*_*t*_ = *P*_*t+*1_ = *H*_*t*_ = *H*_*t*+1_ = 0) were removed from the regression.

To assess the significance of species interactions at each spatial scale, we compared the observed *R*_TD_ and *R*_BU_ values with their corresponding bootstrap null probability distributions. The null probability distribution, median, and the 95% confidence intervals (CI) of *R*_TD_ and *R*_BU_ at each spatial scale were generated by 10,000 resampling iterations. For each resampling iteration *b*, for each time *t*, the resampled population data for patch *i* (denoted *H* ^*(b)*^_*i,t*_) was randomly drawn with replacement from the original data of all patches (e.g., *H* ^*(b)*^_*i,t*_ = *H*_*k,t*_, where *k* is any number from 1 to 81). Aphid and ladybeetle data were resampled independently. We did not analyze data at the largest possible scale (81 plants) because at this scale the null model would be constrained to be very similar to the data.

Because the observed *R*_TD_ and *R*_BU_ in both the experiment and the mathematical model included scale‐irrelevant components, inferences regarding how *R*_TD_ and *R*_BU_ change with spatial scale should be made relative to the median of the null model. For example, synchronized environmental effects (Moran effect; Moran, 1953), phenology, and experimental procedures could produce overall temporal trends in populations for the two species. The gradual decline in aphid population density in the field experiment is an example of this (Appendix S1: Fig. S1). The decreasing predator population through each round was an experimental artifact, but fluctuations in the predator population are common in nature, and the scale‐related pattern should have been robust to the fluctuations. In particular, because the null models included these scale‐irrelevant effects, we could filter them out of the analysis by comparing the observed *R*_TD_ and *R*_BU_ to the null models. To better understand the effect of the decreasing predator population, we conducted two supplementary analyses. First, we analyzed subsets of experimental data produced using two different methods where ladybeetle declines were less severe. Second, we simulated an additional model scenario where ladybeetles could exit the system. Details are described in Appendix S4.

## 3 RESULTS

**Figure 2:**
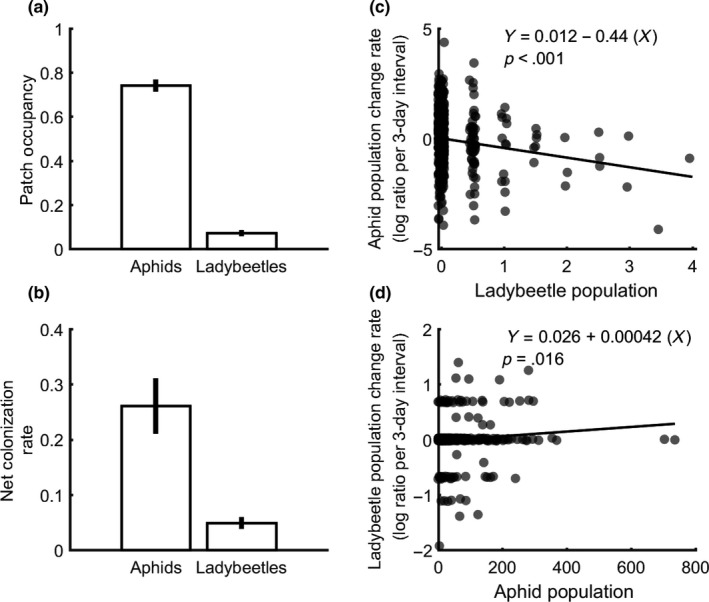
Interaction between ladybeetles and aphids in the field. (a) The patch occupancy. (b) The population colonization rate. Error bars in panels (a)‐(b) are standard errors (SE) over time. (c) The effect of ladybeetles on aphids: The *Uroleucon* aphid population change (log ratio) over a three‐day interval regressed negatively against the average ladybeetle population (adjusted *R* ^2^ = .023; *p* < .001; *n* = 576, slope = −0.44). (d) The effect of aphids on ladybeetles: The ladybeetle population change regressed positively against the aphid population (adjusted *R* ^2^ = .0084; *p* = .012; *n* = 576, slope = −0.00042). In this analysis, adults and larvae of *Cycloneda sanguinea*,* Coccinella septempunctata,* and *Naemia sp*. were lumped as “ladybeetles.”

In the field, aphids were present in many more patches than were ladybeetles (Figure 2a), and the average colonization rate was higher for aphids than for ladybeetles (Figure 2b). Ladybeetles had a strong negative effect on aphid populations at the patch scale (Figure 2c), and aphids had a positive effect on ladybeetles at the patch scale (Figure 2d).

**Figure 3:**
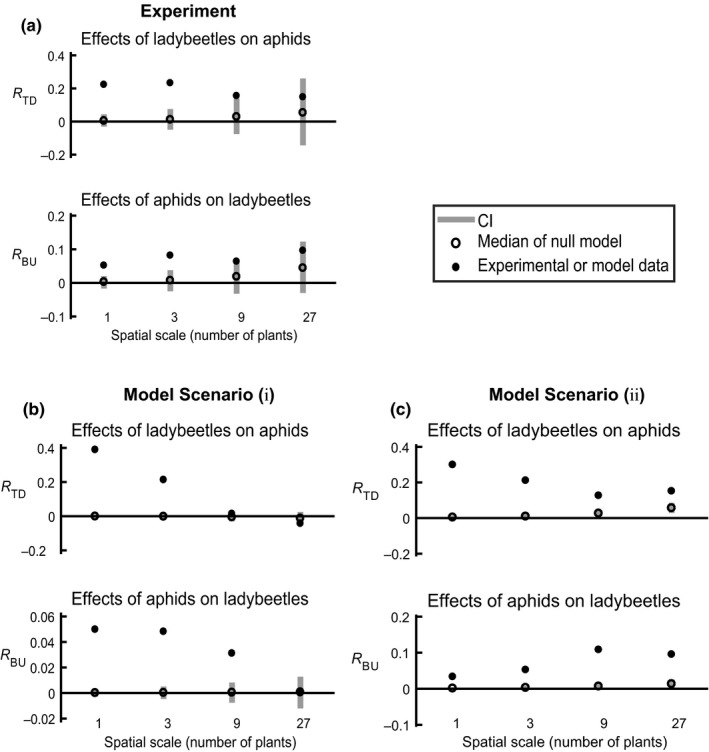
Interactions between ladybeetles and aphids in the experiment and in the model simulations. The solid dots represent the experimental or model simulation data. The gray bars and open circles represent the 95% confidence intervals and medians, respectively, from 10,000 permutations (resampling with replacement) of the null model. (a) Interaction between ladybeetles and aphids calculated from experimental data (*p*‐values of *R*_TD_: <0.0001, <0.0001, 0.014, 0.18; *R*_BU_ : <0.0001, <0.0001, 0.04, 0.10, for 1‐, 3‐, 9‐, 27‐plant scale, respectively). (b) Interaction between ladybeetles and aphids calculated from model simulation scenario (*i*) (without predator growth or death) (*p*‐values of *R*_TD_: <0.0001, <0.0001, 0.06, 0.051; *R*_BU_ : <0.0001, <0.0001, <0.0001, 0.46, for 1‐, 3‐, 9‐, 27‐plant scale, respectively). (c) Interaction between ladybeetles and aphids calculated from model simulation scenario (*ii*) (with predator growth and death) (*p*‐values of *R*_TD_: <0.0001, <0.0001, <0.0001, <0.0001; *R*_BU_ : <0.0001, <0.0001, <0.0001, <0.0001, for 1‐, 3‐, 9‐, 27‐plant scale, respectively)

In the experiment, the effects of ladybeetles on aphids (*R*_TD_) were significant (different from the null model) at the three smaller spatial scales, but not at the largest spatial scale (Figure 3a). The deviation of *R*_TD_ from the null median decreased with increasing spatial scales. The effects of aphids on ladybeetles (*R*_BU_) were significant at the three smaller spatial scales, but not at the largest spatial scale (Figure 3a). The deviation of *R*_BU_ from the null median was highest at the three‐plant scale. Spiders and other species of ladybeetles were present in the experiment but did not qualitatively affect these results (Appendix S3). In the null model, both *R*_TD_ and *R*_BU_ increased with increasing spatial scale (Figure 3a). The deviation of *R*_TD_ and *R*_BU_ values from zero were mostly caused by the overall temporal trends (e.g., in each round of experiments, ladybeetle population size decreased over time (Appendix S1; Fig. S1)). Interpretation of the effects of spatial scale should therefore be based on comparing the observed *R*_TD_ and *R*_BU_ values to the null models. We analyzed subsets of data where the ladybeetle population declines were less pronounced, and the results were qualitatively similar to those obtained from the full dataset (Appendix S4: Fig. S2, S4). We also simulated a model where ladybeetles could exit the system (i.e., decline in abundance), and the model result was qualitatively similar to the experimental result (Appendix S4: Fig. S6). These analyses suggest that the scale‐dependent patterns in Figure 3 were not solely caused by decreasing ladybeetle population.

In the mathematical model scenario (*i*) (without predator demographic processes), both *R*_TD_ and *R*_BU_ decreased with increasing spatial scale and were significantly different from the null model at the two smaller spatial scales (Figure 3b). *R*_TD_ and *R*_BU_ in the null model permutations of the simulation results were all close to zero and had smaller confidence intervals at all spatial scales (Figure 3b) than did the null model of the field experiment, reflecting the larger size of the dataset generated by the mathematical simulations versus that observed in the field experiment. In the mathematical model scenario (ii) (with predator demographic processes), *R*_TD_ decreased, and *R*_BU_ increased with spatial scale from the one‐plant to the nine‐plant scale, but not from the 9‐ to 27‐plant scale. Both *R*_TD_ and *R*_BU_ values were significantly different from the null model at all spatial scales (Figure 3c). The *R*_TD_ and *R*_BU_ in the null models increased slightly with spatial scale (open circles in Figure 3c), suggesting that there were some synchronized fluctuations in the simulated populations.

## 4 DISCUSSION

In the field, we found that even though ladybeetles severely reduced aphid populations when both species were present within a patch, aphids had a much higher colonization rate and occupied many more patches than did ladybeetles. This result implies that ladybeetles had less of an effect on the overall aphid population at the metacommunity scale, likely because the higher population level colonization rate of aphids promoted the persistence of their metapopulation. The population level colonization rate was low for ladybeetles, probably because their population size was much smaller than the aphid population size. These observations suggested that this ladybeetle–aphid system persists at a larger spatial scale as a metacommunity, where colonization–extinction dynamics play an important role in regulating population sizes. Thus, we needed to take a metacommunity approach to understand what happens at these larger spatial scales.

In the experiment, we found that the effect of ladybeetles on aphids (*R*_TD_) was more distinct from the null model at the two smaller spatial scales, and the effect of aphids on ladybeetles (*R*_BU_) was most distinct from the null model at the three‐plant scale. The importance of these results is that they suggest that our interpretation of observed predator–prey dynamics in this system would differ depending on at which spatial scale observations were made. *R*_TD_ and *R*_BU_ were not significantly different from the null models at the larger scales, in part because of the reduced sample sizes, and thus increased confidence intervals, at larger scales (Figure 3a). The pattern for the effects of ladybeetles on aphids was consistent with hypothesis *H1*, that the negative effect of the predator on the prey would decrease with increasing spatial scale. This result suggests that ladybeetles could strongly suppress aphids in a single patch, but could not colonize all the plants fast enough to suppress aphids at larger scales. This result was similar to findings from a crab clam system in which the effects of predation were only significant at smaller spatial scales (Seitz & Lipcius, 2001). We expect this pattern be generalized across systems in which the prey is less mobile than the predator and lacks a spatial refuge. In such systems, predators can strongly suppress the prey within given patches; however, prey persist at larger spatial scales by occupying patches that lack predators for extended periods of time.

In the experiment, the pattern for the effects of aphids on ladybeetles partially supports hypothesis *H2*, that the positive effect of the prey on the predator would decrease with increasing spatial scale. But instead of monotonically decreasing with spatial scale as expected, *R*_BU_ was most divergent from the null model at the three‐plant scale. However, we should be conservative when interpreting results from the two largest scales due to the increased variations. The observed positive relationship between ladybeetle population change and aphid population size at smaller scales was caused by reduced dispersal of ladybeetles in patches with higher aphid densities. Given the relatively short timescale of the experiment, demographic processes were not important: even though 10 clutches of eggs were found, none developed to adults, and including the larva into the analysis did not qualitatively change the result (Appendix S3). We expect a similar pattern to be found in systems where the predator performs area‐restricted searching (or volume‐restricted searching; e.g., Adachi et al., 2017) and prey are less mobile than predators. In such systems, predators can aggregate at patches where prey are abundant. Our results contrast with those from a dragonfly‐tadpole system, where the prey (the tadpole) were mobile and moved to avoid predation. In this case, the predator population were only positively correlated with the prey at larger spatial scales (Hammond, Luttbeg, Brodin, & Sih, 2012).

In model scenario (i) where predators did not have demographic dynamics, we found that both types of species interaction decreased with increasing spatial scale. This result was consistent with our experimental results and was also consistent with hypotheses *H1* and *H2*. As in the experiment, the observed relationship between ladybeetle population change and aphid population size was caused by aggregation of ladybeetles to patches with high aphid densities, with aggregation caused by reduced dispersal from aphid‐rich patches. The qualitative consistency between the experiment and the model suggests that the model captured the most important factors controlling predator–prey interactions for this system, and how these factors varied with spatial scale.

In model scenario (ii) where predators had demographic dynamics, the differences between the effects of ladybeetles on aphids (*R*_TD_) and null model results generally decreased with increasing spatial scale, as predicted in hypothesis *H1*. The effect of aphids on ladybeetles (*R*_BU_) increased with increasing spatial scales. This contradicts hypothesis *H2*. We reasoned that because ladybeetles disperse and feed on multiple patches, their reproduction should be less sensitive to local scale prey density. The two model scenarios correspond to the “behavioral response” and the “intergeneration relationships,” respectively (Hassell, 1966). Our result suggests that the patterns of scale‐dependent species interaction, especially the effects of prey on predators, could be sensitive to whether predator demographic processes are considered. Even in models without predator demographic processes, movement of predators alone can create spatial patterns in predator–prey metacommunities. For example, using partial differential equations, Lewis and Murray (1993) showed that the territorial patterns of wolf packs can be predicted by urination marking and prey‐taxis movements of the wolves (also see White, Murray, & Lewis, 1996). In our case, we show that movement of ladybeetles alone can create scale‐dependent population dynamics patterns.

In our model, we incorporated studies on the movement behavior of herbivorous aphids and predatory ladybeetles, and used this to scale up an understanding of their interactions. The model we used can be viewed as a continuous‐time patch model of a predator–prey metacommunity (Briggs & Hoopes, 2004). By incorporating body size as a parameter into the individual‐based stochastic simulation, our model emulates the ladybeetle–aphid system better than traditional patch dynamic models. For example, body size is negatively correlated to the population size (number of individuals). Population size is important in metacommunity dynamics (Orrock & Watling, 2010), because larger populations are less susceptible to demographic stochasticity and contribute more migrants that can colonize new patches. As we observed in the field, the aphids in the model are not individually more mobile than the ladybeetles, but because aphids are much more abundant, they are better patch colonizers at the landscape level. Also, our model is consistent with field observations that ladybeetles usually leave a patch before all aphids are eaten (e.g. Minoretti & Weisser, 2000). The remaining small population of aphids might then recover without the input of new aphid colonists. In this study, we only simulated the model in scenarios similar to our experimental system. Our results imply that including different body sizes into spatially explicit metacommunity models could produce emergent scale‐related patterns (e.g., Figure 3b,c). The range of possibilities is large and beyond the scope of this study; however, we argue that it would be a promising direction for future efforts.

Despite the consistency in general trends between the experiment and the model scenario lacking predator demography, the results of the experiment and model differed at the smallest spatial scale. In the model, the strongest species interactions were observed at the smallest scale of single plants, but in the experiment, species interactions at this scale were equally or less strong versus those observed at the second‐smallest spatial scale of three‐plant sets. One possible explanation for this discrepancy between model and experiment results is that our model did not explicitly describe how ladybeetles detect plant patches. Because the ability of ladybeetles to detect vegetation is limited to about 1–3 m (Banks & Yasenak, 2003), ladybeetles may have been able to see the other two plants in a three‐plant group before leaving their current plant. This is consistent with previous studies showing that movement of ladybeetles between plants 2 m apart is much greater than when plants are 6 m apart (Grez et al., 2005). Thus, in our experiment, ladybeetles may have perceived the three‐plant sets as the true “patches,” and moved repeatedly among plants within a three‐plant set in a single day. We expect the model would fit the experimental data better if more detailed information about ladybeetle dispersal behavior were included.

Ladybeetles are often viewed as effective biological control agents of aphids, and small‐scale experiments frequently show that they strongly suppress aphid populations (Cardinale, Harvey, Gross, & Ives, 2003; Minoretti & Weisser, 2000; Snyder, Finke, & Snyder, 2008). However, in larger scale field experiments and observations, ladybeetles often fail to control aphid populations (Dixon, 2000; Iperti, 1999; Kindlmann et al., 2015). This discrepancy could be due not only to the spatial aggregation of ladybeetles and aphids associated with spatial scale, as we have shown in this study, but perhaps also to differences in methodology of studies conducted at different spatial scales. In particular, it is likely that the experimental devices used in most small‐scale studies introduced significant artifacts that were not present in the large‐scale studies. For example, cages both prevent dispersal and exclude top predators, which could do as much harm to the ladybeetles as to the aphids (e.g., Ho & Pennings, 2008). A major problem, then, in synthesizing past results across spatial scales is that the effects of scale and the effects of methodology could be hard to disentangle. In our experiment and model, however, we demonstrated a gradual change from the strong predation effects commonly found in small‐scale laboratory/cage studies to the weak effects commonly found in field/landscape studies. Importantly, as we used a consistent methodology as we “scaled up” our observations, our results can be understood as due to spatial scale alone.

The issue of spatial scale presents a significant and under‐appreciated problem for ecology. For the purposes of management and conservation, we usually need information on larger, landscape‐scale patterns. Most experimental studies, however, are performed at small (Englund & Cooper, 2003) and often rather arbitrary (Wheatley & Johnson, 2009) scales, and thus, if not scaled up properly might not provide the best insight into the problems that they were intended to address. Studies in other systems have shown that predator–prey interactions and abundance patterns vary with observational scale (e.g., bivalves: Seitz & Lipcius, 2001; fishes: Rose & Leggett, 1990; seabirds and fish: Fauchald et al., 2000). Spatially explicit models have also demonstrated that the population dynamics of a predator–prey metacommunity can be scale dependent (e.g., De Roos, Mccauley, & Wilson, 1991). However, to the best of our knowledge, the present study is the first using parallel experimental and model approaches to study the scale dependency of predator–prey interactions. We argue that a better and more general understanding of how ecological processes vary with spatial scale would help bridge the gap between typical ecological experiments conducted at small scales and ecological problems that often are at the landscape scale, and we encourage more studies that explicitly incorporate analyses at a range of spatial scales.

## CONFLICT OF INTEREST

None declared.

## DATA ACCESSIBILITY

Field observation data are publically available from the LTER network data portal, https://doi.org/10.6073/pasta/fae8e94a866b71fad5e9122136b5b95e (Lin, 2016). Data from the field experiment are provided in Appendix S1. MATLAB code for reproducing the model is available online in Zenodo, https://doi.org/10.5281/zenodo.268964 (Lin, 2017).

## AUTHOR CONTRIBUTIONS

SP and WL conceived the ideas and designed field methodology; WL collected and analyzed the data; WL constructed and simulated the model; SP and WL led the writing of the manuscript. All authors contributed critically to the drafts and gave final approval for publication.
