# Supplementary material for: Predator–prey interactions in a ladybeetle–aphid system depend on spatial scale
Source: Ecol Evol. 2018 Jun 11;8(13):6537–46. doi: 10.1002/ece3.4117 (PMC6053568; doi:10.1002/ece3.4117)
Supplement: Supplementary file 1 [file ECE3-8-6537-s001.docx]

**Appendix S1: Overall (81-plant scale) population trends in the experiment, and experimental data (1-plant scale)**

There were overall temporal trends in populations of aphids and ladybeetles in the experiments because of phenology and experimental procedures. In each round of the experiment, ladybeetles tended to disperse out of the system, creating a decline in ladybeetle population density within each round. The gradual decline in aphid population density over the entire course of the experiment may be caused by seasonal phenology.


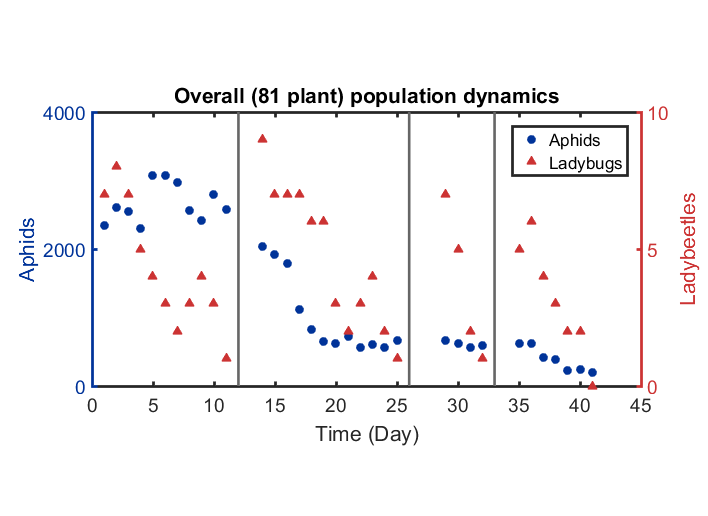


**Appendix S1: Figure S 1** Ladybeetle populations are plotted on the secondary axis. The gray lines separated four rounds of experiment, where ladybeetles were restocked. Day 1 was May 17, 2014.


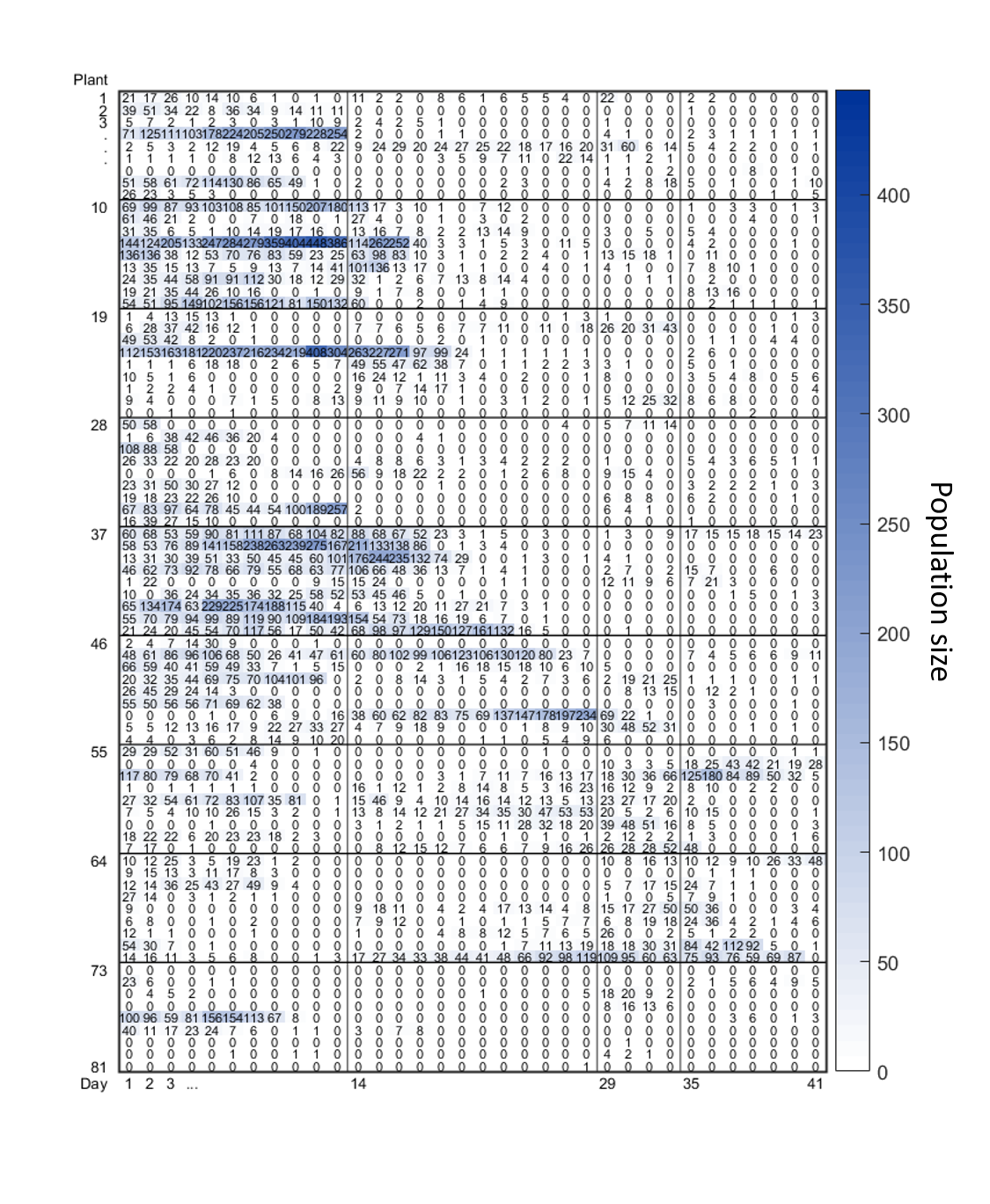


**Appendix S1: Figure S 2** Aphid population size on each plant in the field experiment. Each row is the time series of aphid population size on a single plant; each column is a single day. Cells in the table are color-coded as a function of aphid density, with the density key on the right side of the table. The horizontal lines group the individual plants into 9-plant sets. Within each 9-plant set, the plants were grouped consecutively into 3-plant sets (e.g., plants 1, 2, 3 were a 3-plant set). Similarly, the 9-plant sets were grouped consecutively into 27-plant sets. The vertical lines separate the four rounds of the experiment, where ladybeetles were restocked. Day 1 was May 17, 2014.


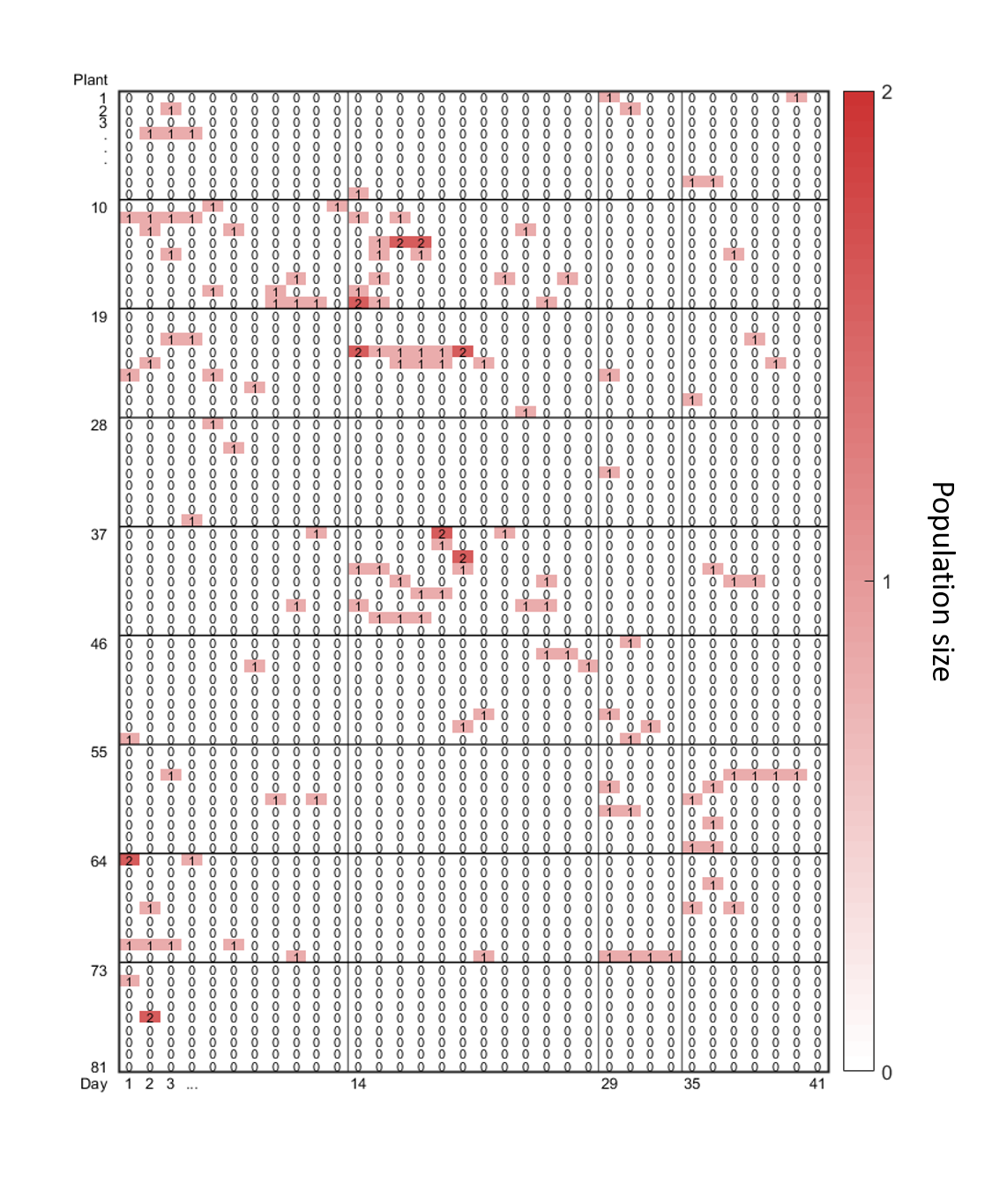


**Appendix S1: Figure S 3** Ladybeetle population sizes on each plant in the field experiment. Data are organized as in Figure S 2.
