# Supplementary material for: Predator–prey interactions in a ladybeetle–aphid system depend on spatial scale
Source: Ecol Evol. 2018 Jun 11;8(13):6537–46. doi: 10.1002/ece3.4117 (PMC6053568; doi:10.1002/ece3.4117)
Supplement: Supplementary file 2 [file ECE3-8-6537-s002.docx]

**Appendix 2: Details of the mathematical model**

***Simulation Landscape***

The model was spatially explicit, with a landscape comprised of 81 patches arranged in a triangular array as in the experimental system. The distance between two patches within a 3-plant set was 2 units, and the distance between the centers of two 3-plant sets within a 9 plant set was 6 units, and so on, to maintain the same spatial structure as in the field experiment.

***Dispersal functions***

***Emigration***


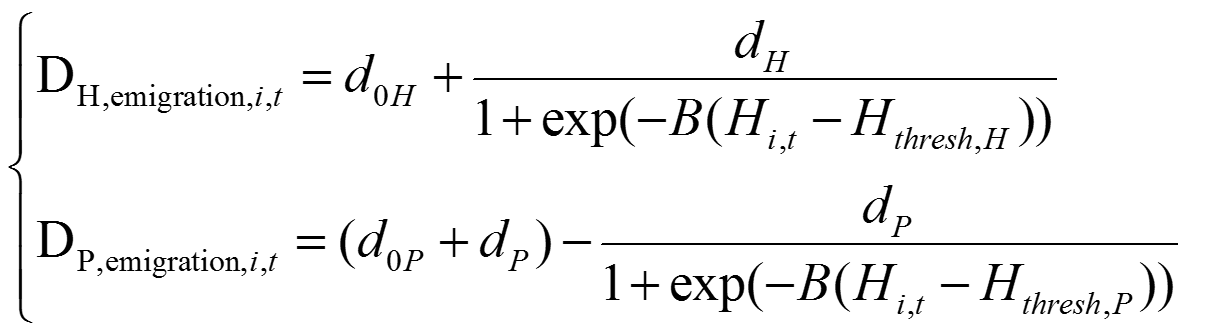
The dispersal rate (the tendency of emigration for each individual) for ladybugs (the predator) and aphids (the herbivore) were functions of herbivore population density. For aphids, we assumed the emigration rate only depended on crowding. The effect of predators on aphid wing polymorphism is weak compared to crowding (Purandare, Tenhumberg & Brisson 2014), even though it has been documented in some species (Dixon & Agarwala 1999; Weisser, Braendle & Minoretti 1999).

Where *d_0H_* is the background emigration rate of the herbivore, and *d_H_* is the increase in emigration rate when herbivore density is much higher than the crowding threshold, (*H_i,t_* >> *H_thresh,H_*). Similarly, *d_0P_* is the background emigration rate of the predator, and *d_P_* is the increase in emigration rate when prey are rare (*H_i,t_* << *H_thresh,P_*). Parameter B controls how steep the logistic function is (smaller value means the transition is more smooth).


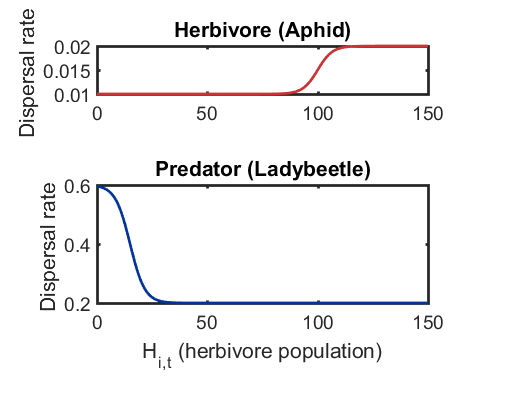


**Appendix S2: Figure S 1** The relationship between herbivore population size and the per capita emigration rates of the herbivore and the predator.

***Settlement***

The emigrants settled on other patches randomly according to the incidence function. The relative settlement rate depended on the distance between the origin and destination patches. The probability that each emigrant *X* (*X* represents *H* or *P*) from patch *i* will settle at patch *j* was modeled as an incidence function (Hanski & Woiwod 1993): exp(-*c_Xv_ d_i,j_*) / ( Σ*_s_* exp( -*c_X_* *d_i,s_*) ), where *d_i,j_* is the distance between patch *i* and *j*, and *c_X_* is a parameter for the dispersal kernel, with small values representing long-distance dispersal. Similar equations have been previously used to model aphid and moth dispersal (Hanski & Woiwod 1993).


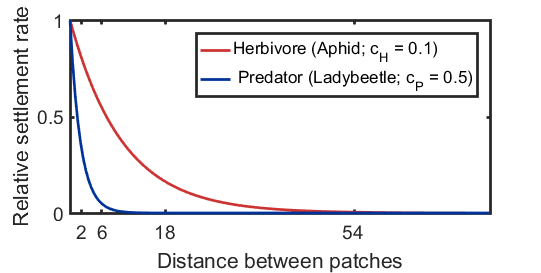


**Appendix S2: Figure S 2** The relationship between the distance between patches and the relative probability of settlement according to the incidence function.

***Gillespie’s algorithm***

To simulate the model with Gillespie’s algorithm, we first rewrote equation 1 in terms of the number of individuals. We assumed the body size of predator and herbivore individuals to be *S_P_* and *S_H_*, and we denoted the number of predator and herbivore individuals as *N_Pi,t_* and *N_Hi,t_*, respectively, where *N_Pi,t_* = *P_i,t_*/*s_P_, N_Hi,t_ = H_i,t_*/*s_H_*. Then we rescaled the model so *s_H_* = 1. Then, for each of the 81 patches, there were six possible events: birth, death, and dispersal of the two species. Assuming these events are Poisson stochastic events, the birth, death and dispersal rates are:

1. *Birth of herbivore: g_H_ N_H_*
2. *Death of herbivore: (g_H /_ K_H_ ) N_H_^2^ + (e_P_ / ( N_H_ + H_0_ )) N_H_ N_P_ s_P_ +m_H_ N_H_*
3. *Birth of predator: a_P_ (e_P_ / (N_H_ + H_0_ )) N_H_ N_P_*
4. *Death of predator: m_P_ N_P_*
5. *Emigration of herbivore:* D_H,emigration_ *N_H_*
6. *Emigration of predator:* D_P,emigration_ *N_P_*

For dispersal events (event (5) and (6)), we assumed that each emigrant immediately settled on a patch according to the incidence function. For model scenario (*i*) we assumed that the birth and death rates of the predator were zero.

**Appendix S2: Table S 1**

| **Parameter** | **Meaning** | **Default** |
| --- | --- | --- |
| ***g_H_*** | The intrinsic growth rate of the herbivore | 0.2 |
| ***a_P_*** | The assimilation rate of the predator | 0.2 |
| ***k_H_*** | The carrying capacity of the herbivore | 200 |
| ***e_P_*** | The consumption rate of the herbivore by the predator | 3 |
| ***H_0_*** | The half saturation density of the herbivore to the predator | 30 |
| ***m_H_*** | The density-independent mortality rate of the herbivore | 0.1 |
| ***m_P_*** | The density-independent mortality rate of the predator | 0.05 |
| ***d_H_*** | The increased emigration rate of the herbivore when crowded (*H_i,t_* >= *H_thresh,H_*) | 0.01 |
| ***d_0H_*** | The background emigration rate of the herbivore | 0.01 |
| ***H_thresh,H_*** | The threshold of herbivore density of being crowded | 100 |
| ***c_H_*** | The parameter for herbivore dispersal incidence  (small number means long-distance dispersal) | 0.1 |
| ***d_P_*** | The increased emigration rate of the predator when food is scarce (*H_i,t_* <= *H_thresh,P_*) | 0.4 |
| ***d_0P_*** | The background emigration rate of the predator | 0.2 |
| ***H_thresh,P_*** | The threshold of herbivore density to induce higher predator emigration rate. | 15 |
| ***c_P_*** | The parameter for predator dispersal incidence | 0.5 |
| ***B*** | The smoothing parameters of the dispersal function | 0.3 |
| ***sH*** | The body size of the herbivore | 1 |
| ***sP*** | The body size of the predator | 20 |

**References**

Dixon, A.F.G. & Agarwala, B.K. (1999) Ladybird-induced life–history changes in aphids. *Proceedings of the Royal Society of London B: Biological Sciences,* **266,** 1549-1553.

Hanski, I. & Woiwod, I.P. (1993) Spatial Synchrony in the Dynamics of Moth and Aphid Populations. *Journal of Animal Ecology,* **62,** 656-668.

Purandare, S.R., Tenhumberg, B. & Brisson, J.A. (2014) Comparison of the wing polyphenic response of pea aphids (Acyrthosiphon pisum) to crowding and predator cues. *Ecological Entomology,* **39,** 263-266.

Weisser, W.W., Braendle, C. & Minoretti, N. (1999) Predator-induced morphological shift in the pea aphid. *Proceedings of the Royal Society of London Series B-Biological Sciences,* **266,** 1175-1181.
