# Supplementary material for: Predator–prey interactions in a ladybeetle–aphid system depend on spatial scale
Source: Ecol Evol. 2018 Jun 11;8(13):6537–46. doi: 10.1002/ece3.4117 (PMC6053568; doi:10.1002/ece3.4117)
Supplement: Supplementary file 3 [file ECE3-8-6537-s003.docx]

**Appendix S3: The effects of spiders, other ladybeetle species and larva in the experiment**

***The effects of spiders***


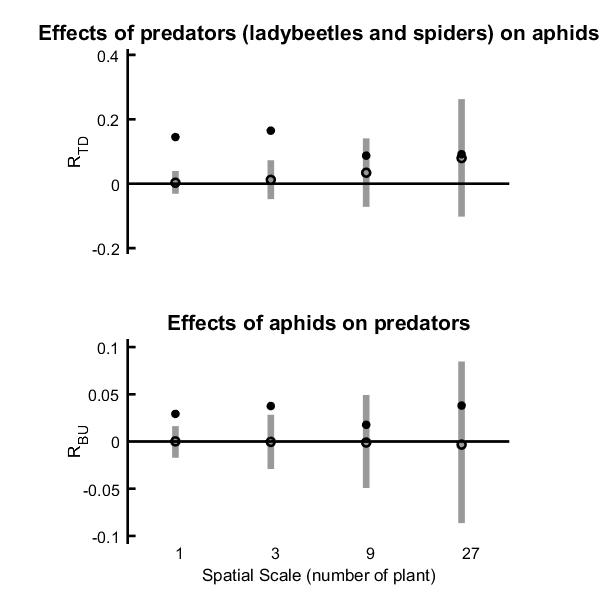
There were 194 records (individuals * days) of spiders in the experiment, and 141 records of ladybeetles of our focal species (*Cycloneda sanguinea*). Common spiders in this system don’t eat aphids (Marczak *et al.* 2011). Pooling spiders as predators with ladybeetles did not qualitatively change the results.

**Appendix S3: Figure S 1**

***The effects of other ladybugs***


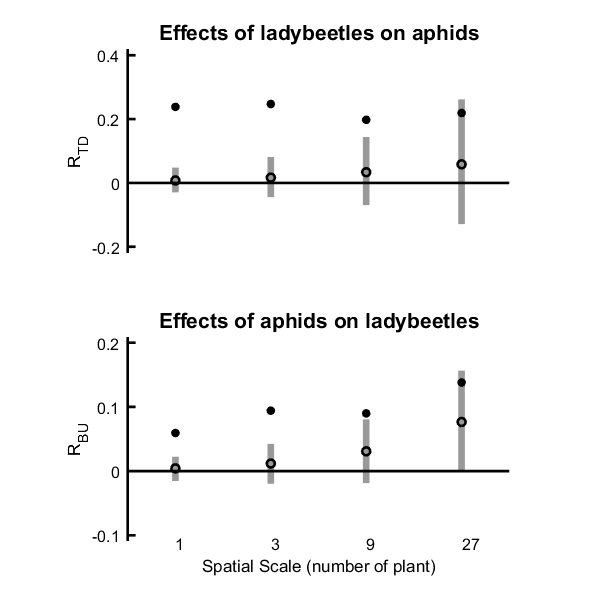
There were 27 occurrences of ladybeetles of species other than *Cycloneda* (Coccinella septempunctata and *Naemia sp.*). Pooling other ladybeetles with *Cycloneda* did not qualitatively change the results.

**Appendix S3: Figure S 2**

***The effects of ladybeetle larva***

During the experiment 10 clutches of ladybeetle eggs were recorded, 4 of which hatched. Larval numbers rapidly declined, presumably due to cannibalism or emigration, and none of the larvae pupated on the plants. Moreover, we did not observe larvae successfully dispersing to other plants. Given a typical *Cycloneda sanguinea* ladybeetle life cycle (4.0 days for eggs to hatch; 8.6 days for larvae to develop; 4.7 days for pupation; at 25。C; Cardoso and Lázzar 2003), and that the longest stretch of our experimental rounds was 12 days, egg clutches were unlikely to affect ladybeetle adult numbers. Thus, the effect of ladybeetle larvae on aphids was limited to only four plants, and reproduction had no detectable effect on adult ladybeetle numbers.

**Literature cited**

Cardoso, J.T. & Lázzar, S.M.N. (2003) Comparative biology of Cycloneda sanguinea (Linnaeus, 1763) and Hippodamia convergens Guérin-Méneville, 1842 (Coleoptera, Coccinellidae) focusing on the control of Cinara spp.(Hemiptera, Aphididae). *Revista Brasileira de Entomologia,* **47,** 443-446.

Marczak, L.B., Ho, C.-K., Wieski, K., Vu, H., Denno, R. & Pennings, S. (2011) Latitudinal variation in top-down and bottom-up control of a salt marsh food web. *Ecology,* **92,** 276-281.
