# Supplementary material for: Predator–prey interactions in a ladybeetle–aphid system depend on spatial scale
Source: Ecol Evol. 2018 Jun 11;8(13):6537–46. doi: 10.1002/ece3.4117 (PMC6053568; doi:10.1002/ece3.4117)
Supplement: Supplementary file 4 [file ECE3-8-6537-s004.docx]

**Appendix S4: The effects of decreasing ladybeetle populations**

We conducted two additional analyses to better understand the effect of decreasing ladybeetle populations throughout each round of the experiment. First, we analyzed subsets of experimental data produced using two different methods where ladybeetle population declines were less severe. Second, we simulated a special model scenario where ladybeetles could exit the system.

***Analyzing subsets of experimental data***

We created a subset of the experimental dataset by removing days where the overall ladybeetle population declined for two consecutive days. The trimmed subset of data contained 23 days consisting of 7 continuous segments (Appendix S4: Figure S1). When we analyzed these data, the resulting scale-dependent pattern was similar to that obtained from the full dataset (Appendix S4: Figure S2).


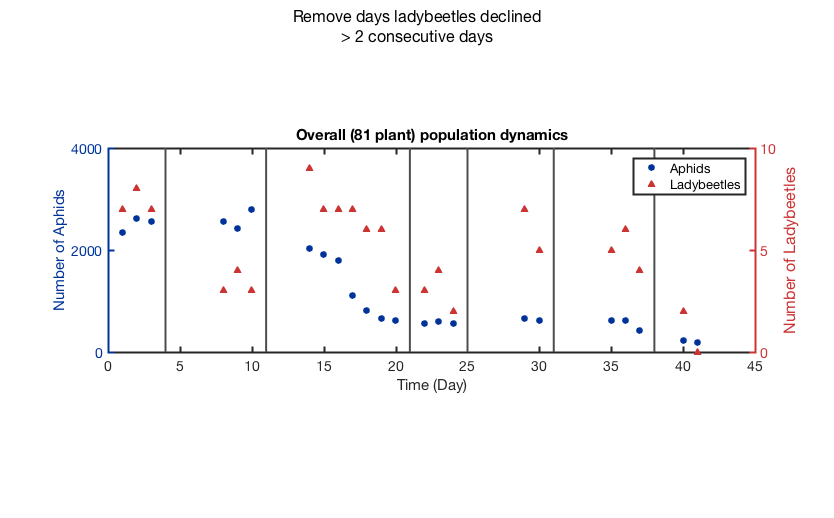


**Appendix S4: Figure S 1**

***
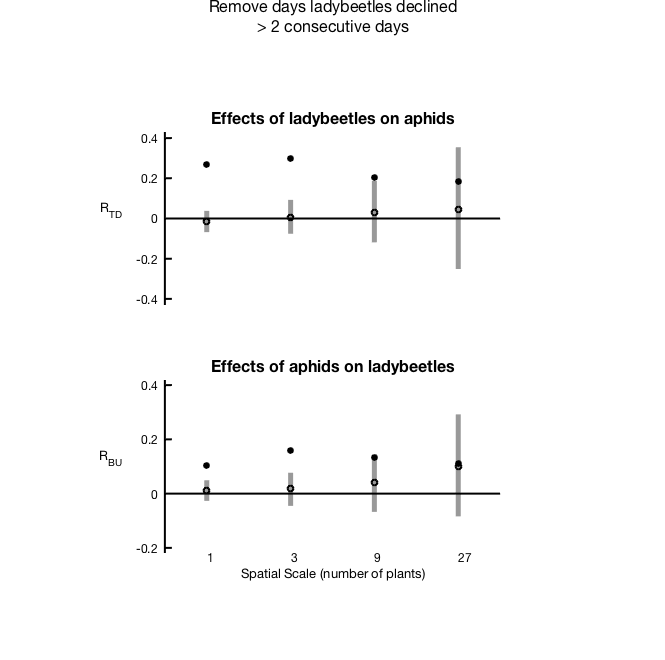
***

**Appendix S4: Figure S 2**

We created another subset of the experimental dataset, by removing days where the overall ladybeetle population was smaller than three. This goal of this trimming process was to reduce the importance of very small ladybeetle populations at the end of each experimental round. The trimmed subset of data contained 24 days of 6 continuous segments (Appendix S4: Figure S3). When we analyzed these data, the resulting scale-dependent pattern was again similar to that obtained from the full dataset (Appendix S4: Figure S4).

***
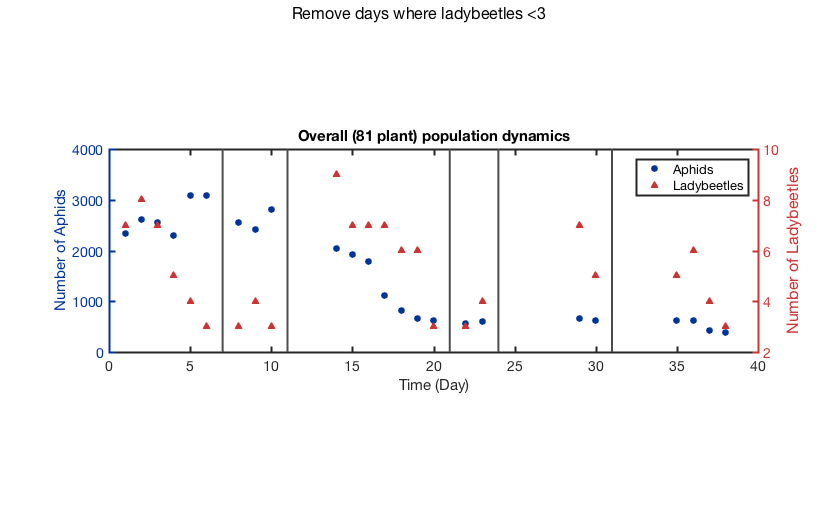
***

**Appendix S4: Figure S 3**

***
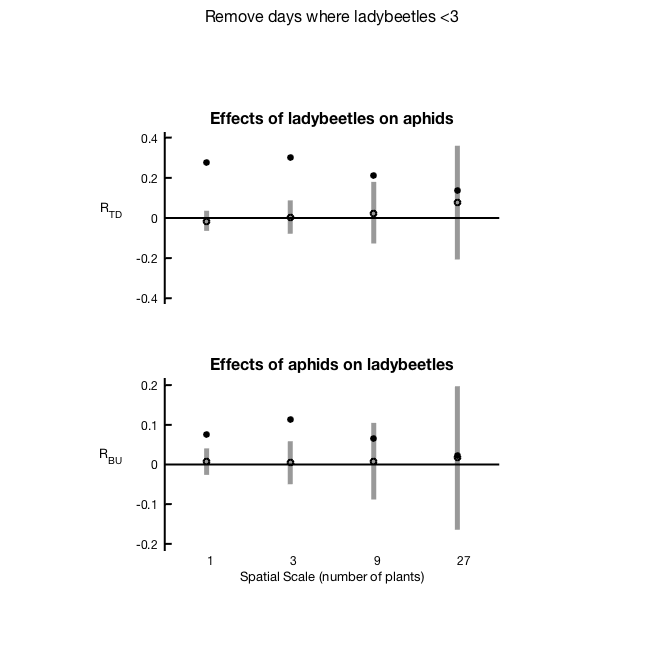
***

**Appendix S4: Figure S 4**

***Simulating the effect of reducing ladybeetle populations***

We simulated an additional model scenario where ladybeetle population decreased in each round of simulation due to ladybeetles exiting the experimental system. We added a parameter, *exit_P_* = 0.05, to describe the rate with which a dispersing ladybeetle does not settle on any patch but instead “exits” the system. We also changed the simulation procedure to obtain non-trivial data: we initialized ladybeetle population size to three times that of the default to compensate for the decline, and recorded populations at t = 21~30 (instead of 91~100). In this way, we recorded “transient” population dynamics as in the experiment. Other parameters were the same as model scenario (*i*). Appendix S4: Figure S5 shows the first ten rounds of simulation.

The model with ladybeetle declines (Appendix S4: Figure S6) fit our experimental results better, while the model without ladybeetle declines (main text: Figure 3b) showed clearer scale-dependent patterns and fit the hypotheses better. This comparison demonstrated the potential effect of declining ladybeetle populations, and implies that the deviation between our experiment and the hypothesized patterns might be partly caused by the declining ladybeetle populations.


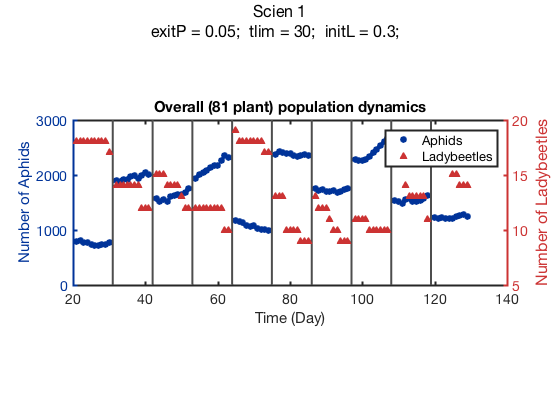


**Appendix S4: Figure S 5**


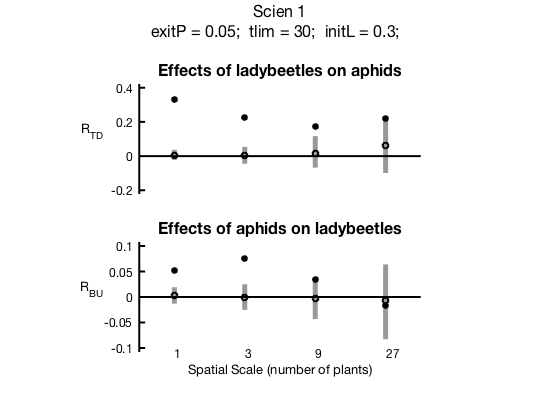

**Appendix S4: Figure S 6**
